# PIEZO1 force sensing in cardiac electrical modelling

**DOI:** 10.1113/JP291882

**Published:** 2026-09-17

**Authors:** Yurou Cai, Michael A Colman, David J Beech

**Affiliations:** 1 Leeds Institute of Cardiovascular and Metabolic Medicine, School of Medicine, University of Leeds, Leeds, UK, LS2 9JT; 2 School of Biochemical Sciences, University of Leeds, Leeds, UK, LS2 9JT

## Introduction

Electrical control of myocardial contraction is fundamental to heart function. It involves integrating ionic mechanisms such as voltage-gated Ca^2+^ channels for coordinated rhythms and cytosolic Ca^2+^ elevations. Many aspects have been modelled computationally for health and disease simulations with considerable success and predictive value. The heart is, however, also highly mechanical. Ogiermann et al make an important effort to integrate this mechanical component in electrical modelling for mechano-electric feedback (Ogiermann *et al*., 2026). They rightly address the challenge by focusing on the PIEZO1 channel. While not the only force sensor in the heart, PIEZO1 is an especially well defined force sensor at protein structural and functional levels and widely accepted by investigators as dedicated to mechanical force sensing (Xiao, 2024). Its discovery was recognized by the Nobel Prize for Physiology or Medicine in 2021. It is expressed and functional in myocardium and other cardiac structures (Beech, 2026). It is a Ca^2+^-permeable non-selective cationic channel that singularly and rapidly couples mechanical force to transmembrane Ca^2+^, Na^+^ and K^+^ fluxes, regulating both membrane potential and cytosolic Ca^2+^ concentration potentially on the millisecond timescale (Xiao, 2024).

Integrating PIEZO1 requires consideration of its dynamic gating properties. Its mechanical activation is rapid, within milliseconds, and the switching off (deactivation) when mechanical force dissipates is almost as rapid. There is also inactivation conferring adaptive desensitization to continuous mechanical force and refractoriness to repeated mechanical stimuli. This type of deep closure is similar in principle to inactivation in voltage-gated ion channels. The inactivation is a key regulated property of PIEZO1 such that it may occur quickly over about 100 milliseconds or more slowly over seconds or minutes. This seems to be a way the same force sensor, PIEZO1, confers sensing in diverse mechanical and cell contexts. One of several modulators of the inactivation is voltage, with a shift to slower inactivation at positive voltages. It remains largely to be determined how PIEZO1 is adapted in cardiac cells such as myocytes.

### Study outline

A PIEZO1 model was developed with closed, open, fast-inactivated and slow-inactivated states of the channel, adapting an earlier model and incorporating state transitions depending on mechanical and electrical driving forces (Ogiermann *et al*., 2026). It reproduced features of PIEZO1, generating rapidly activating currents followed by inactivation. It captured voltage dependence and outward rectification. With repeated stimulation, there was expected desensitization at negative voltages and pressure-dependent resetting at positive voltages.

The PIEZO1 model was then incorporated in a rabbit ventricular cardiomyocyte model as a mechanically activated, non-selective cation current of the t-tubular system detached from the dyadic cleft (Ogiermann *et al*., 2026). With pacing, PIEZO1’s activation increased intracellular Na^+^ and Ca^2+^ concentrations, depressed the action potential waveform and shortened the action potential duration. Brief mechanical pulses generated sufficient inward current to depolarize the cardiomyocyte and initiate action potentials, demonstrating mechanical capture. With repeated mechanical stimulation there was progressive loss of capture, consistent with more PIEZO1 channels entering inactivated states. Although multiple parameters were tested, the model could not reproduce a fast loss of capture during alternating stimuli nor fully account for frequency dependence.

## Discussion

The study provides a compact channel-to-cell framework for starting to evaluate how mechanical- and voltage-dependent gating of PIEZO1 could contribute to cardiac electromechanical pacing (Ogiermann *et al*., 2026). It brings advances by combining a voltage-modulated PIEZO1 gating formulation with ventricular electrophysiology, connecting electrically and mechanically induced excitation in ways that can be refined and tested experimentally.

The model is a phenomenological representation of macroscopic PIEZO1 activity rather than a one-on-one mapping to distinct physical conformations of the channel, which are still known only quite superficially at the protein structural level. Although the inclusion of fast- and slow-inactivated states in the model is supported by multi-exponential inactivation and frequency-dependent responses, these states have not been mapped independently to defined structural conformations. Modelling in the article should therefore be regarded as a functional kinetic hypothesis rather than a demonstrated molecular gating mechanism. This level of simplification is reasonable and appropriate given the available experimental constraints. Additional states and transition pathways might improve the apparent fit but would also introduce more parameters than the existing macroscopic current data justify.

The multiple pressure- and voltage-dependent rate parameters were derived from experimental data for PIEZO1 channel currents measured in mouse neuroblastoma (N2A) cells. Simulation data of the article qualitatively reproduce the voltage dependence of these currents and rectification, but the model was not accurate in all regards including inactivation rate. Because the voltages at which there was mismatch overlap those of the resting and subthreshold range of ventricular cardiomyocytes, there could be overestimation of PIEZO1-evoked depolarization.

Modelling and its parameterization have important implications for physiological relevance. Data from symmetric K^+^ conditions informing the model differ substantially from the mixed ionic environment of cardiomyocytes. The fitted dependence on electrical driving force may therefore not transfer quantitatively to native cardiac conditions. More broadly, the model transfers kinetic parameters across species and cell type from mouse PIEZO1 and the mouse N2A cells to rabbit PIEZO1 and the rabbit ventricular myocyte. To the best of our knowledge, the properties of rabbit PIEZO1 channels and mechanically activated PIEZO1 channels of cardiac myocytes of any species are unknown. It therefore remains uncertain whether mouse N2A PIEZO1 data accurately represent native PIEZO1 of rabbit heart. Recordings from myocardial fibroblasts have suggested relatively slowly inactivating PIEZO1 channels currents in cardiac cells (Jakob *et al*., 2021). The precision of kinetic estimates is likely to be crucial in models of cardiac electrical patterns.

The model also depends on uncertain assumptions about PIEZO1 expression levels, membrane density and maximal conductance. Because an initial PIEZO1-mediated depolarization can recruit voltage-gated Na^+^ and Ca^2+^ channel currents, mechanical capture is an emergent property of the integrated cardiomyocyte model rather than a direct consequence of PIEZO1 activity alone. The model therefore suggests PIEZO1-like current can initiate mechanical capture under the selected conductance-scaling assumptions, but it remains undetermined whether native ventricular PIEZO1 is sufficient under physiological conditions. Experimental data that would be helpful for modelers includes PIEZO1 ionic current recordings from freshly isolated rabbit ventricular myocytes under physiological ion, temperature and mechanical conditions.

The inability of a single parameter set to reproduce all pacing protocols suggests that PIEZO1 state occupancy alone may not account for capture and capture loss. There are therefore limits to the present formulation and opportunities for refinement. An amplification pathway that could be incorporated in future models is functional coupling between PIEZO1 channels and TRPM4 channels, which are Ca^2+^-activated but Na^+^-selective channels (Yu *et al*., 2022). Experimental data quantifying the contribution of downstream ionic mechanisms in rabbit ventricular myocytes in physiological conditions would likely improve the accuracy of the modelling.

Also important to consider in future models are the feedback loops of the intact myocardium from muscle contraction and mechano-transduction to Ca^2+^ dynamics and electrical excitation (Izu *et al*., 2020). Contraction-induced changes in membrane mechanics may modify subsequent PIEZO1 activation or recovery. More advanced models may successfully embed PIEZO1 in such coupled, multiscale electromechanical frameworks linking cellular electrophysiology, Ca^2+^-dependent active tension, tissue deformation and local membrane tension.

Nevertheless, within its context, this new modelling illustrates the balance required between biological detail, parameter identifiability and interpretability (Ogiermann *et al*., 2026). Given the available protein structural and macroscopic current data, substantially increasing model complexity would risk introducing poorly constrained parameters without a commensurate gain in mechanistic insight. The four-state formulation, by contrast, remains compact, thermodynamically constrained and calibrated against published experimental currents. Its primary value lies in testing whether a defined PIEZO1-mediated mechanism can generate mechanically induced cardiac excitation under selected assumptions. We believe this initial step made is valuable and promising for future studies building on the model. The conditions under which the model fails to reproduce experimental observations are informative because they delineate the explanatory limits of a PIEZO1-centred, single-cell mechanism and illustrate the need for better experimental data on the properties of PIEZO1 channels in cardiac myocytes.

## Supplementary Material

Peer Review History
