# Supplementary material for: PIEZO1 force sensing in cardiac electrical modelling
Source: J Physiol. Author manuscript; Available in PMC 2026 Oct 16. (PMC7619564; doi:10.1113/JP291882)
Supplement: Supplementary file 1 [file EMS218570-supplement-Peer_Review_History.pdf]

The Journal of Physiology

Due 31 July: Journal Club on JP-RP-2025-288666R2 (Ogiermann et al) PIEZO1 force sensing in cardiac electrical modelling

Yurou Cai, Michael A Colman, and David J. Beech
DOI: 10.1113/JP291882

Corresponding author(s): Yurou Cai (umyc@leeds.ac.uk)

The referees have opted to remain anonymous.

Review Timeline:

Submission Date:	31-Jul-2026
Editorial Decision:	12-Aug-2026
Revision Received:	17-Aug-2026
Accepted:	24-Aug-2026

Senior Editor: Bjorn Knollmann

Reviewing Editor: Axel Loewe

Transaction Report:

(Note: With the exception of the correction of typographical or spelling errors that could be a source of ambiguity, letters and reports are not edited. Depending on transfer agreements, referee reports obtained elsewhere may or may not be included in this compilation. Referee reports are anonymous unless the Referee chooses to sign their reports.)

Dear Dr Cai,

Re: JP-JC-2026-291882 "Due 31 July: Journal Club on JP-RP-2025-288666R2 (Ogiermann et al) **PIEZO1 force sensing in cardiac electrical modelling**" by Yurou Cai, Michael A Colman, and David J. Beech

Thank you for submitting your manuscript to The Journal of Physiology. It has been assessed by a Reviewing Editor and by 1 expert referee and we are pleased to tell you that it is acceptable for publication following satisfactory revision.

Please share this decision with your co-authors as soon as possible.

The referee reports are copied at the end of this email.

Please address all the points raised and incorporate all requested revisions or explain in your Response to Referees why a change has not been made. We hope you will find the comments helpful and that you will be able to return your revised manuscript within 2 weeks. If you require longer than this, please contact journal staff: jp@physoc.org.

Your revised manuscript should be submitted online using the link in your Author Tasks Link Not Available. This link is accessible via your account as Corresponding Author; it is not available to your co-authors. If this presents a problem, please contact journal staff (jp@physoc.org). Image files from the previous version are retained on the system. Please ensure you replace or remove any files that are being revised.

If you do not wish to submit a revised version of your manuscript, you must inform our journal staff (jp@physoc.org) or reply to this email to request withdrawal. Please note that a manuscript must be formally withdrawn from the peer review process at one journal before it may be submitted to another journal.

TRANSPARENT PEER REVIEW POLICY: To improve the transparency of its peer review process The Journal of Physiology publishes online, as supporting information, the peer review history of all articles accepted for publication. Readers will have access to decision letters, including Editors' comments and referee reports, for each version of the manuscript, as well as any author responses to peer review comments. Referees can decide whether or not they wish to be named on the peer review history document.

LANGUAGE EDITING AND SUPPORT FOR PUBLICATION: If you would like help with English language editing, or other article preparation support, Wiley Editing Services offers expert help, including English Language Editing, as well as translation, manuscript formatting, and figure formatting at www.wileyauthors.com/eeo/preparation. You can also find resources for Preparing Your Article for general guidance about writing and preparing your manuscript at www.wileyauthors.com/eeo/prepresources.

REVISION CHECKLIST:

Upload a full Response to Referees file. To create your 'Response to Referees' copy all the reports, including any comments from the Senior and Reviewing Editors, into a Microsoft Word, or similar, file and respond to each point, using font or background colour to distinguish comments and responses and upload as the required file type.

Please upload two versions of your manuscript text: one with all relevant changes highlighted and one clean version with no changes tracked. The manuscript file should include all tables and figure legends, but each figure/graph should be uploaded as separate, high-resolution files. The journal is now integrated with Wiley's Image Checking service. For further details, see: https://www.wiley.com/en-us/network/publishing/research-publishing/trending-stories/upholding-image-integrity-wileys-image-screening-service

You may also upload:
- 'Potential Cover Art' for consideration as the issue's cover image
- Appropriate Supporting Information (Video, audio or data set: see https://jp.msubmit.net/cgi-bin/main.plex?form_type=display_requirements#supp).

We look forward to receiving your revised submission.

If you have any queries, please reply to this email and we will be pleased to advise.

Yours sincerely,

Bjorn Knollmann
Senior Editor
The Journal of Physiology

REQUIRED ITEMS

- Please include a full, separate title page as part of your main article (Word) file, which should contain the following: title, authors, affiliations, corresponding author name and contact details, keywords, and running title.

- Authors are required to fully disclose the use of any Generative AI (GAI) tools used in the preparation of their manuscript. This disclosure should also be included as a statement alongside the Additional Information and Data Availability statements towards the end of the article under the heading 'Generative AI Statement', and include the name(s), model and version of AI technology used, and exact details on the purpose for, and methods of, its use.

If no AI tool has been used, this should also be specifically stated within the manuscript.

EDITOR COMMENTS

Reviewing Editor:

Thank you for your Journal Club contribution, which was reviewed positively. The referee's suggestion to be more specific about the experimental data provides opportunity to further enhance the value of your manuscript.

Senior Editor:

I agree with the reviewing editor. In the revision, please give details about which experimental data specifically could be helpful for modelers to inform their efforts

----

REFEREE COMMENTS

Referee #1:

The authors present a well-written Journal Club article critically reviewing a recent paper from Ogiermann et al. about a novel Piezo1 Markov Chain model as an electromechanical force sensor and its integration into an established rabbit cardiomyocyte model (Mahajan-Shiferaw). They appreciate the central role of Piezo1 in mechano-electrical and mechano-chemical feedback and highlight the need for future modeling efforts. They elaborate on the models' limitations to open up the path for subsequent modeling and point at the need for more experimental data on Piezo1 channels in cardiomyocytes. The line of argumentation is sound and covers a wide range of relevant strenghts and weaknesses about the study. My only minor critique point on the article is that it misses a great opportunity to give details about which experimental data specifically could be helpful for modelers to inform their efforts.

END OF COMMENTS

JP-JC-2026-291882 R1

Author responses to editor and referee.

Re: JP-JC-2026-291882 "Due 31 July: Journal Club on JP-RP-2025-288666R2 (Ogiermann et al) PIEZO1 force sensing in cardiac electrical modelling" by Yurou Cai, Michael A Colman, and David J. Beech

Thank you for submitting your manuscript to The Journal of Physiology. It has been assessed by a Reviewing Editor and by 1 expert referee and we are pleased to tell you that it is acceptable for publication following satisfactory revision.

Thank you.

Please share this decision with your co-authors as soon as possible.

All authors received a copy and contributed to the revision.

The referee reports are copied at the end of this email.

Please address all the points raised and incorporate all requested revisions or explain in your Response to Referees why a change has not been made. We hope you will find the comments helpful and that you will be able to return your revised manuscript within 2 weeks. If you require longer than this, please contact journal staff: jp@physoc.org.

We responded fully with changes to the manuscript: "JP-JC-2026-291882 R1". Clean and track-changes versions are provided.

Your revised manuscript should be submitted online using the link in your Author Tasks https://jp.msubmit.net/cgi-bin/main.plex?el=A2JS5IPl4A1mFT1F1A9ftdhTYdax1ZtnOgQ3YuEcWQDQZ. This link is accessible via your account as Corresponding Author; it is not available to your co-authors. If this presents a problem, please contact journal staff (jp@physoc.org). Image files from the previous version are retained on the system. Please ensure you replace or remove any files that are being revised.

If you do not wish to submit a revised version of your manuscript, you must inform our journal staff (jp@physoc.org) or reply to this email to request withdrawal. Please note that a manuscript must be formally withdrawn from the peer review process at one journal before it may be submitted to another journal.

We submitted a revision.

TRANSPARENT PEER REVIEW POLICY: To improve the transparency of its peer review process The Journal of Physiology publishes online, as supporting information, the peer review history of all articles accepted for publication. Readers will have access to decision letters, including Editors' comments and referee reports, for each version of the manuscript, as well as any author responses to peer review comments. Referees can decide whether or not they wish to be named on the peer review history document.

LANGUAGE EDITING AND SUPPORT FOR PUBLICATION: If you would like help with English language editing, or other article preparation support, Wiley Editing Services offers expert help, including English Language Editing, as well as translation, manuscript formatting, and figure formatting at www.wileyauthors.com/eeo/preparation. You can also find resources

for Preparing Your Article for general guidance about writing and preparing your manuscript at www.wileyauthors.com/eeo/prepresources.

REVISION CHECKLIST:

Upload a full Response to Referees file. To create your 'Response to Referees' copy all the reports, including any comments from the Senior and Reviewing Editors, into a Microsoft Word, or similar, file and respond to each point, using font or background colour to distinguish comments and responses and upload as the required file type.

We uploaded a point-by-point response (this file).

Please upload two versions of your manuscript text: one with all relevant changes highlighted and one clean version with no changes tracked. The manuscript file should include all tables and figure legends, but each figure/graph should be uploaded as separate, high-resolution files. The journal is now integrated with Wiley's Image Checking service. For further details, see: https://www.wiley.com/en-us/network/publishing/research-publishing/trending-stories/upholding-image-integrity-wileys-image-screening-service

We uploaded one version with all relevant changes highlighted and one clean version with no changes tracked.

You may also upload:
- 'Potential Cover Art' for consideration as the issue's cover image
- Appropriate Supporting Information (Video, audio or data set: see https://jp.msubmit.net/cgi-bin/main.plex?form_type=display_requirements#supp).

We do not propose cover art.

We look forward to receiving your revised submission.

Thank you for your consideration. We look forward to your response and would be happy to respond to any further suggestions.

If you have any queries, please reply to this email and we will be pleased to advise.

Yours sincerely,

Bjorn Knollmann
Senior Editor
The Journal of Physiology

REQUIRED ITEMS

- Please include a full, separate title page as part of your main article (Word) file, which should contain the following: title, authors, affiliations, corresponding author name and contact details, keywords, and running title.

We included a full, separate title page as suggested.

- Authors are required to [<a]https://jp.msubmit.net/cgi-bin/main.plex?form_type=display_requirements#aigcfully disclose the use of any Generative

AI (GAI) tools used in the preparation of their manuscript. This disclosure should also be included as a statement alongside the Additional Information and Data Availability statements towards the end of the article under the heading 'Generative AI Statement', and include the name(s), model and version of AI technology used, and exact details on the purpose for, and methods of, its use.

If no AI tool has been used, this should also be specifically stated within the manuscript.

We added a declaration that “No AI tools were used”.

EDITOR COMMENTS

Reviewing Editor:

Thank you for your Journal Club contribution, which was reviewed positively. The referee's suggestion to be more specific about the experimental data provides opportunity to further enhance the value of your manuscript.

Thank you. We took the opportunity to improve the manuscript as suggested and specified below.

Senior Editor:

I agree with the reviewing editor. In the revision, please give details about which experimental data specifically could be helpful for modelers to inform their efforts

Thank you. We added details about which experimental data could be helpful for modelers.

REFEREE COMMENTS

Referee #1:

The authors present a well-written Journal Club article critically reviewing a recent paper from Ogiermann et al. about a novel Piezo1 Markov Chain model as an electromechanical force sensor and its integration into an established rabbit cardiomyocyte model (Mahajan-Shiferaw). They appreciate the central role of Piezo1 in mechano-electrical and mechano-chemical feedback and highlight the need for future modeling efforts. They elaborate on the models' limitations to open up the path for subsequent modeling and point at the need for more experimental data on Piezo1 channels in cardiomyocytes. The line of argumentation is sound and covers a wide range of relevant strenghts and weaknesses about the study. My only minor critique point on the article is that it misses a great opportunity to give details about which experimental data specifically could be helpful for modelers to inform their efforts.

Thank you.

We added details about which experimental data could be helpful for modelers, as follows:

“Experimental data that would be helpful for modelers includes PIEZO1 ionic current recordings from freshly isolated rabbit ventricular myocytes under physiological ion, temperature and mechanical conditions.” Lines 118-120.

"Experimental data quantifying the contribution of downstream ionic mechanisms in rabbit ventricular myocytes in physiological conditions would likely improve the accuracy of the modelling." Lines 127-129.

We also made two minor corrections to our originally submitted text. Lines 20 and 81.

We added a declaration that no AI tools were used.

END OF COMMENTS

Dear Dr Cai,

Re: JP-JC-2026-291882R1 "Due 31 July: Journal Club on JP-RP-2025-288666R2 (Ogiermann et al) **PIEZO1 force sensing in cardiac electrical modelling**" by Yurou Cai, Michael A Colman, and David J. Beech

We are pleased to tell you that your paper has been accepted for publication in The Journal of Physiology.

The last Word (or similar) version of the manuscript provided will be used by the Production Editor to prepare your proof. When this is ready you will receive an email containing a link to Wiley's Online Proofing System. The proof should be thoroughly checked and corrected as promptly as possible.

Authors should note that it is too late at this point to offer corrections prior to proofreading. Major corrections at proof stage, such as changes to figures, will be referred to the Editors for approval before they can be incorporated. Only minor changes, such as to style and consistency, should be made at proof stage. Changes that need to be made after proof stage will usually require a formal correction notice.
All queries at proof stage should be sent to: TJP@wiley.com.

Following copyediting and typesetting the accepted version will be published online.

Yours sincerely,

Bjorn Knollmann
Senior Editor
The Journal of Physiology

IMPORTANT POINTS TO NOTE FOLLOWING ACCEPTANCE OF YOUR PAPER:

• IMPORTANT NOTICE ABOUT OPEN ACCESS: To assist authors whose funding agencies mandate immediate public access to published research findings, The Journal of Physiology allows authors to pay an Open Access (OA) fee to have their papers made freely available immediately on publication.

The Corresponding Author will receive an email from Wiley with details on how to register or log in to Wiley Authors where you will be able to place an order.

You can check if your funder or institution has a Wiley Open Access Account here:
https://authors.wiley.com/author-resources/Journal-Authors/open-access/author-compliance-tool.html

MEMBER DISCOUNT: Corresponding authors who are Members of the Physiological Society are eligible for a 20% discount on the cost of the Article Processing Charge (APC). To claim this discount, the Corresponding Author will need to log in to the Physiological Society Membership portal and retrieve the discount code (https://www.physoc.org/journals-and-media/journals/journal-discount-for-members/). This code should then be quoted in the billing section of Wiley Authors when selecting the open access option.

• TRANSPARENT PEER REVIEW POLICY: To improve the transparency of its peer review process, The Journal of Physiology publishes online as supporting information the peer review history of all articles accepted for publication. Readers will have access to decision letters, including Editors' comments and referee reports, for each version of the manuscript as well as any author responses to peer review comments. Referees can decide whether or not they wish to be named on the peer review history document.

• You can help your research get the attention it deserves! Check out Wiley's free Promotion Guide for best-practice recommendations for promoting your work at: www.wileyauthors.com/eeo/guide. You can learn more about Wiley Editing Services which offers professional video, design, and writing services to create shareable video abstracts, infographics, conference posters, lay summaries, and research news stories for your research at: www.wileyauthors.com/eeo/promotion.

• If you would like to receive our 'Research Roundup', a monthly newsletter highlighting the cutting-edge research published in The Physiological Society's family of journals (The Journal of Physiology, Experimental Physiology, Physiological Reports, The Journal of Nutritional Physiology, and The Journal of Precision Medicine: Health and Disease), please click this link, fill in your name and email address and select 'Research Roundup':
https://www.physoc.org/journals-and-media/membernews

EDITOR COMMENTS

Reviewing Editor:

Thank you for improving the manuscript according to the referee's suggestions.

Senior Editor:

The MS is acceptable. Thank you for your contribution!
